# The p-medicine portal—a collaboration platform for research in personalised medicine

**DOI:** 10.3332/ecancer.2014.398

**Published:** 2014-02-11

**Authors:** Fatima Schera, Gabriele Weiler, Elias Neri, Stephan Kiefer, Norbert Graf

**Affiliations:** 1 Fraunhofer IBMT, Ensheimer Strasse 48, 66386 St. Ingbert, Germany; 2 CUSTODIX, Kortrijkseteenweg 214 b3, 9830 Sint-Martens-Latem, Belgium; 3 Universitätsklinikum des Saarlandes, Kirrberger Straße, 66424 Homburg, Germany

**Keywords:** personalized medicine, p-medicine portal, enterprise portal framework Liferay, portlet, integration, deployment, p-medicine tools and services, data mining, ontology, ObTiMA

## Abstract

The European project p-medicine creates an information technology infrastructure that facilitates the development from current medical practice to personalised medicine. The main access point to this infrastructure is the p-medicine portal that provides clinicians, patients, and researchers a platform to collaborate, share data and expertise, and use tools and services to improve personalised treatments of patients. In this document, we describe the community-based structure of the p-medicine portal and provide information about the p-medicine security framework implemented in the portal. Finally, we show the user interface and describe the p-medicine tools and services integrated in the portal.

## 1. Introduction

In the European Union project p-medicine, an information and communications technology infrastructure that facilitates the translation from current practice to personalised medicine has been created. Within p-medicine, new tools including virtual physiological human models are developed to path the way to personalised medicine. These tools will support clinicians in the decision-making process for individualised treatments that will result in better health care for patients. A user-friendly common access point to these tools and services is realised via a portal of the integrated p-medicine platform.

In this article, we describe the architecture of the p-medicine portal and provide information on how the p-medicine security framework is implemented in the portal. Finally, we show the user interface and describe the p-medicine tools and services integrated in the portal.

## 2. Using the Liferay framework in p-medicine

The p-medicine portal is a single web-based environment from which most of the p-medicine applications can run. These applications are integrated in a consistent and systematic way in the form of a web-based user interface.

As a technical solution of the p-medicine portal, we have selected the enterprise portal framework Liferay (http://www.liferay.com). Using the unique constructs the platform provides, we designed a site that can handle any situation needed for the p-medicine portal. The Liferay framework enables users to build web pages and websites; it integrates over 60 portlet applications, which cover about all of the standard functionalities the p-medicine users are likely to need in a website: content management, forums, wikis, blogs, and so on. It provides an advanced user management and security solution.

The Liferay framework allows administrators and end users to build web pages without writing program code by reusing the existing ‘portlets’, which are pluggable user interface components that are managed and displayed in a web portal. To enable interoperability of portlets Java Portlet specifications, Java Specification Request (JSR) 168 (http://jcp.org/aboutJava/communityprocess/final/jsr168/) and JSR 286 (http://www.jcp.org/en/jsr/detail?id=286) are used as standards.

The portal provides role-based content delivery: the portal websites display different data depending on a user’s role. p-medicine uses a powerful paradigm given by Liferay for organising users and giving them access to the content they want to see. We can use communities, organisations, roles, and user groups to make sure that the right content gets to the right people and that restricted content is protected as only the proper users can view it.

The described functionality is built in to the Liferay framework. p-medicine users can pick the applications and drop them onto their pages. Liferay provides a multitude of tools and utilities for increasing developer productivity. That helped us to implement the features specific to the p-medicine project more efficiently.

## 3. Portal users and the community-based structure of the portal

At its most basic level, the p-medicine portal has users, and these users can be grouped together in various ways, providing a powerful mechanism for the portal administrator to configure portal resources and security in a consistent and robust manner. Based on discussions with end users, we have identified and defined the p-medicine portal user groups and their roles in the portal. However, in the future, new roles may arise. Different roles giving permissions for using different tools developed under p-medicine can be created dynamically.

In p-medicine, we have decided to use the community-based structure of the portal. This means that portal users belong to communities that have a common interest (e.g., different portal users are members of a community called *SIOP Nephroblastoma Community* that has a common interest in nephroblastoma research). Portal users can join and leave communities whenever they want. Users can share data and content collected in repositories available for the community members.

Membership in communities gives users access to the pages in the communities of which they are members. Each community can have a specific layout and its own set of available pages containing different p-medicine tools.

Communities for specific domains (e.g., breast cancer) or specific user collections (patients, scientists, and developers) can be created in the portal dynamically.

There are two kinds of roles: portal and community roles. Roles are used to define permissions across their scopes: across the portal or across a community. Each community in the portal has its own configured permissions, so that membership in different communities results in different roles.

## 4. User interface of the p-medicine portal

**Figure 1.**
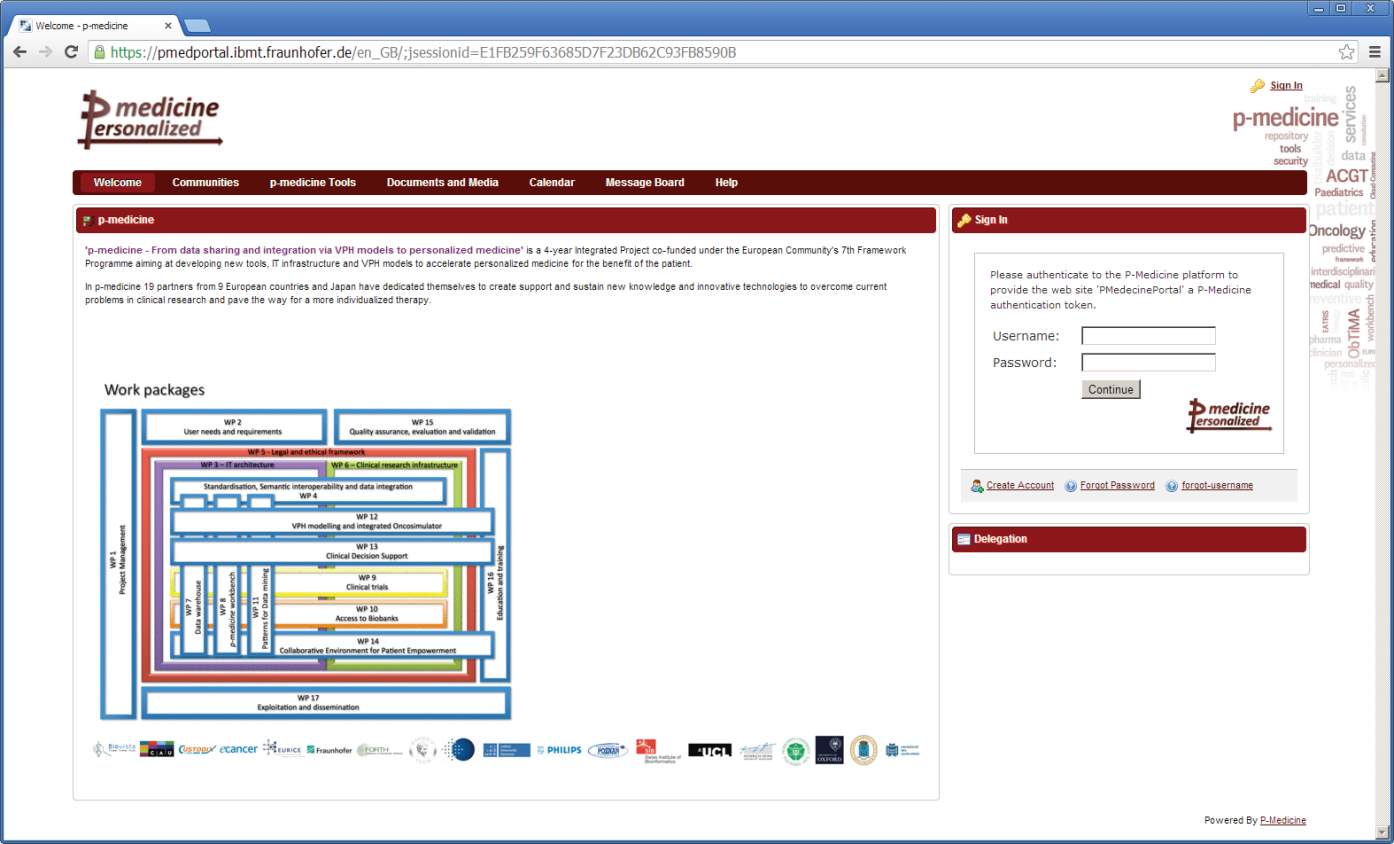
The start page of the p-medicine portal.

For p-medicine, we have developed a plugin for the portal responsible for the p-medicine look and feel, which has replaced the original Liferay layout. The start page of the p-medicine portal is shown in Figure 1.

**Figure 2.**
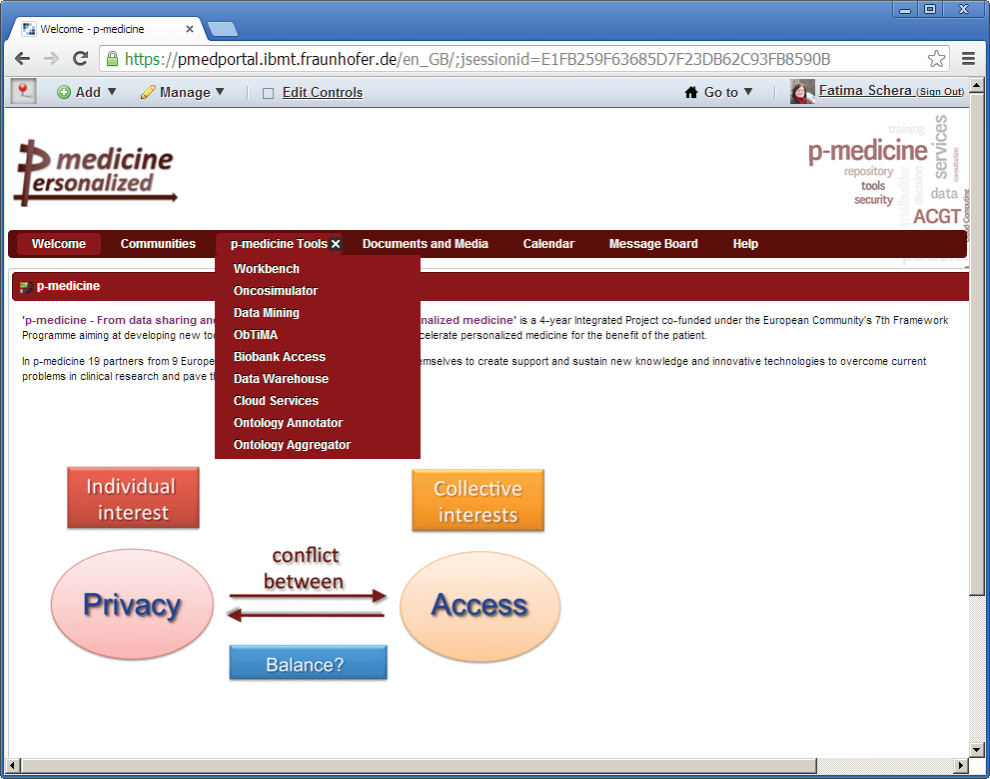
The main p-medicine navigation menu.

The main navigation menu for the p-medicine users contains the following pages:

- ‘Welcome’—a page with the information about the p-medicine project. The *Login* portlet is shown on the right side of the page (Figure 1). Here, more portlets can be placed (e.g., with important announcements for the p-medicine users). This page is visible to everybody (including guests).
- ‘Communities’—a page with (a) a list of communities where the user is member and (b) with the available user communities. According to the community-based structure of the p-medicine portal, the users can participate or leave available communities.
- ‘p-medicine Tools’—this is a set of pages for p-medicine tools and services (Figure 2) included as portlets on the pages to perform the p-medicine scenarios.

Some portlets provided by the Liferay framework, which can be used by the p-medicine users, are also included on the portal pages, e.g.:

- ‘Documents and Media’ portlet for managing p-medicine documents and media content (pictures, video, audio files, etc) according to their permissions.
- ‘Calendar’ and ‘Message Board’ portlet where authorised users can manage events and announcements for displaying to the portal users according to their permissions.

### 4.1 Template site for creation of a default p-medicine community

A new community can be dynamically created in the portal. A community administrator manages the community membership and defines a layout and content of the community pages in the portal. Creating content with a large amount of applications available in the p-medicine portal is a time-intensive task. In order to make this work more comfortable and time effective for the community administrator, we have developed a template for the standard p-medicine community with the predefined layout and contents. It includes a set of pages containing p-medicine-related content. The template can be used as a basis for a newly created community and it will take less time to edit the already included content or to add additional pages as to create the new community site from scratch.

**Figure 3.**
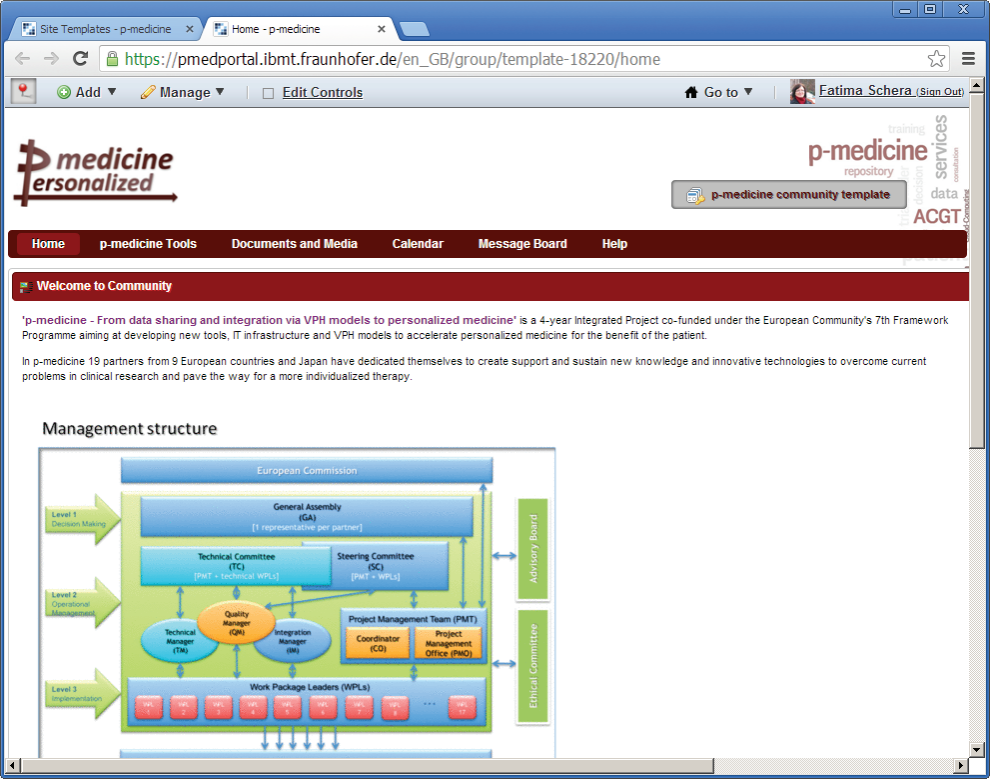
The p-medicine community template view.

On the top of the template page, the name of the template is displayed: the text ‘p-medicine community template’ on the place of the community name (Figure 3) indicates that this is a template, not a community site.

**Figure 4.**
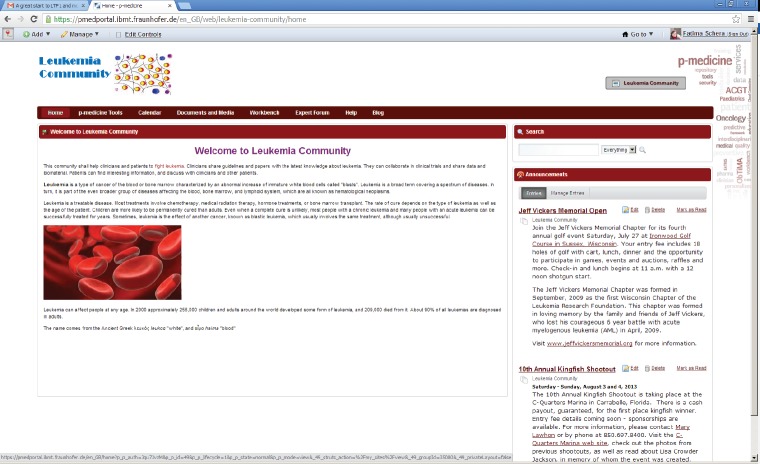
The community for acute lymphoblastic leukaemia.

If the portal administrator selects this template as a primary structure during the creation of a new community, the new community will be created with the content provided in the template. The community administrator can easily adjust the default content according to the community purposes: he can modify the layout of the community site pages; he can add new pages and applications as well as remove some pieces of the content if they are not necessary for the community. He can also replace the default p-medicine logo with the logo representing the community, e.g., in Figure 4, an example community for acute lymphoblastic leukaemia is shown.

### 4.2 Dockbar

After a login, the portal displays the Dockbar, the second user navigation menu at the top of the page, which gives the user access to several functions provided by the Liferay framework. The scope of the visible menu item depends on the user role in the portal.

Here is a short explanation of some of the Dockbar’s menu components:

- ‘Add’—using this option, a fully searchable categorised view of all the applications that have been installed in the portal and that can be added to a page is shown. The p-medicine applications also appear in this list, e.g., data-mining tools.
- ‘Manage’—this option is used to manage pages, page layouts, and so on. One can group pages in the order one wishes and also to nest them into subpage levels. A user can also apply layouts to communities and single pages. Finally, if the user is a community administrator, he can change the logo for a community.
- ‘Go To’—this option is used to navigate to the various community pages to which the user has access and to the control panel, which is the central location where just about everything can be administered:
  - A portal user can manage his own personal space, e.g., change the account information and manage his own personal pages.
  - A community administrator has an access to content management functions. He can maintain web content, documents, images, bookmarks, and a calendar; administer a message board; configure a wiki; and so on.
  - The portal administrators can set up and maintain the portal. This is where he can add and edit users, organisations, communities, and roles as well as configure the settings of the portal.

## 5 p-medicine tools and services

The p-medicine portal aggregates the set of portlets representing the p-medicine tools that are to appear on any particular page and displays them properly to the p-medicine users. The portal administrator arranges the p-medicine tools on the portal’s pages in the way that works best for the portal users.

There are the domain specific tools and services integrated in the portal **directly** as plugins and tools that are used as parts of other tools accessed from the portal for performing user workflows, which are therefore **indirectly** integrated into the portal.

We expect that the list of the tools and services will be extended in the future and the newly developed tools and services will be integrated into the p-medicine environment dynamically.

For integration, some p-medicine portlets have been developed using the Plugin Development Toolkit (http://www.liferay.com/) provided by Liferay (e.g., p-medicine Workbench—see Section 5.2). These portlets have been added seamlessly to the portal’s pages in a way that is indistinguishable from the built-in portlets.

Some p-medicine services are provided as a programmatic interface for using as parts of other tools. They can be semantically annotated and registered in the tools repository and can be presented in the p-medicine workbench portlet in the portal.

The p-medicine tools available as external web applications (e.g., Ontology Annotator) have been integrated using an *iframe* portlet, which makes it possible to embed another Hypertext Markup Language (HTML) page inside the current page.

Below, we show an approach for semantic integration of heterogeneous data sources as well as the p-medicine tools, their functionality, and integration in the portal.

### 5.1 Health Data Ontology Trunk as a semantic basis for data integration and sharing in p-medicine

In p-medicine, there is a need to gain access to clinical data stored in different data sources, such as hospital information systems (HIS) and clinical trial management systems (CTMS), in a transparent way, following appropriate pseudonymisation and security procedures. It is of outmost importance to harmonise the data semantically in this process, in order that the semantics of the data is clearly defined, a precondition to reuse the data for reliable analysis and simulations.

The approach in p-medicine to solve these problems is a newly developed semantic reference for p-medicine’s purposes, the Health Data Ontology Trunk (HDOT) [1]. HDOT ontology is the model that acts as a central schema in the data integration infrastructure, it harmonises and links the existing semantic resources (e.g., ontologies and terminologies), such as Logical Observation Identifier Names (http://loinc.org/), Gene Ontology (http://www.geneontology.org/), and Medical Dictionary for Regulatory Activities (http://www.meddra.org/), under one umbrella. It also integrates resources, which are widely applied and well known by medical staff, as e.g., ICD-10 (http://www.who.int/classifications/icd/en/). The provision for allowing the use of such terms and codes makes HDOT user friendly. In particular, HDOT provides a machine-readable semantic background for these resources. That means that as opposed to many existing resources, HDOT has underlying machine-readable axioms and supports automated reasoning.

This approach will foster the secondary usage of health information system (HIS) and CTMS data for various use cases important to realise personalised medicine, as e.g., simulations, data mining, and the reuse of HIS data in the ontology-based CTMS ObTiMA. Details of p-medicine’*s* approach for semantic data integration are described in [2].

### 5.2 p-medicine Workbench

**Figure 5.**
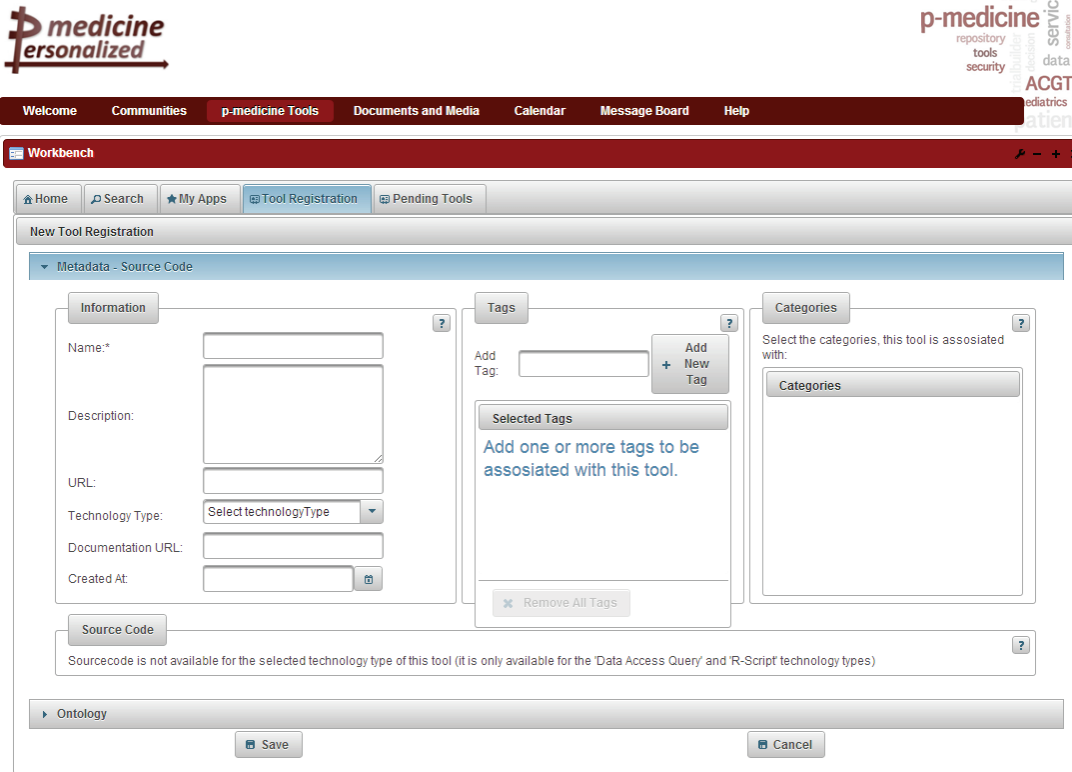
The p-medicine Workbench.

The p-medicine Workbench is an application that aggregates the p-medicine tools and services and provides to the end users various management possibilities for publishing, discovering, evaluating, combining, and invoking these tools. Clinicians are able to use these tools that lack a specific user interface or that are not integrated into the portal framework through the p-medicine Workbench in order to expedite various tasks of their daily practice. The tools and services of p-medicine are registered in a tool repository, where an archive is kept of their metadata and annotations, quality characteristics, documentation, and invocation endpoint. The p-medicine Workbench application is already integrated as a portlet into the portal (Figure 5).

### 5.3 ObTiMA

ObTiMA (http://obtima.org/) is an ontology-based clinical trial management web application intended to support clinicians in both designing and conducting clinical trials [3]. The trial builder in which all aspects of a clinical trial can be specified facilitates the design phase. A graphical user interface allows defining content, navigation, and layout of ontology-based clinical record forms (CRFs) developed by a trial chairman to capture all patient data during a trial, e.g., medical findings or diagnostic data.

ObTiMA also integrates push services that are able to push selected data into the data warehouse. The data and images uploaded to the data warehouse are anonymised by Custodix Anonymisation Tool Services (CATS; see Section 5.12) before storing it into the data warehouse.

ObTiMA includes a trial biomaterial manager—one of the main parts of the biobank access framework. The trial biomaterial manager enables management of biomaterial data in clinical trials and sharing selected biomaterial data.

As a module of ObTiMA, the sync services will be developed to allow reusing of data stored in the electronic clinical records in HIS in clinical trials in ObTiMA. The retrieved data are stored in the CRF of a running clinical trial in ObTiMA. This shall partially avoid entering data manually in CRFs when they are already included in the HIS. The sync services will retrieve the data not directly from the HIS but from the p-medicine data warehouse, since this data are already integrated compliant to HDOT.

**Figure 6.**
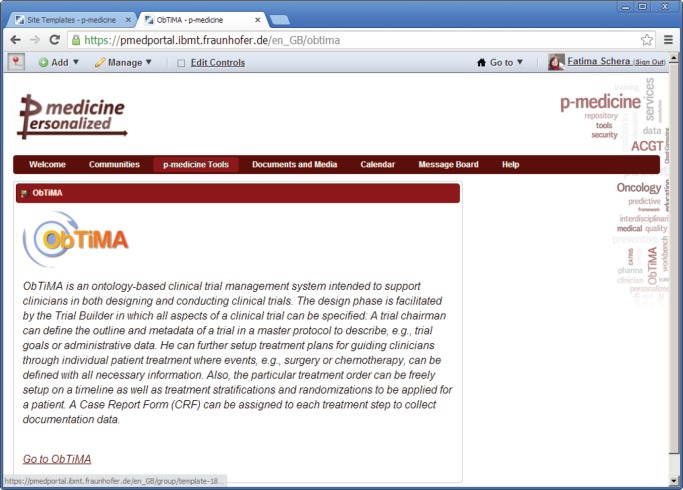
The web link to the ObTiMA application.

ObTiMA is already integrated as a simple web link included in a web content shown in the portal (Figure 6).

Because the single sign-on functionality is implemented in the portal, the user does not need to login into ObTiMA again since the credentials are forwarded from the portal to ObTiMA.

### 5.4 Biobank access framework

**Figure 7.**
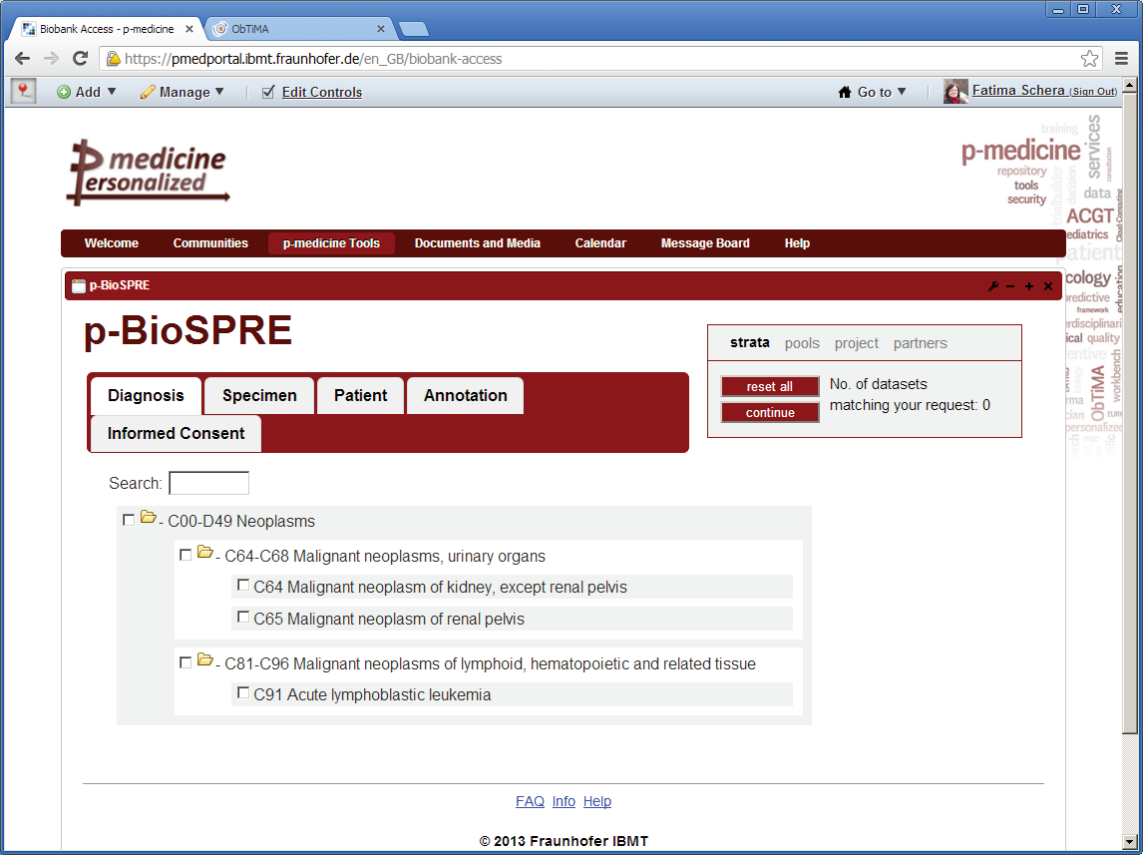
p-BioSPRE.

The p-medicine biobank access framework provides access to different kinds of human biomaterials and related data for research purposes. The p-medicine biobank access framework contains the following three main components:

**p-BioSPRE,** the p-medicine Biomaterial Search and Project Request Engine that is a metabiobank for providing researchers the possibility to search for and request biomaterial that is offered within his communities and which fits their research purposes. p-BioSPRE is a web application already integrated in the p-medicine portal in an iframe container (Figure 7). The application’s management of user roles and rights is compliant to the p-medicine security framework (Section 6).

**p-Biobank wrappers** are tools to support biobank owners to offer their biomaterial and related data within an open or closed research community, which can be stored in any biobank information system, in p-BioSPRE, and that manage associated requests to order biomaterial. Furthermore, a p-biobank wrapper comprises push services, which enable a biobank owner to push selected biomaterial data into the p-medicine data warehouse (see Section 5.3) and share them for the integration with other biomedical data sources. These services will be available in the project later.

**ObTiMA trial biomaterial manager** has been developed as a component of ObTiMA to enable users of ObTiMA to manage biomaterial data in clinical trials and to share selected biomaterial data (see Section 5.5). This component is already integrated in ObTiMA.

### 5.5 Oncosimulator

The p-medicine oncosimulator has been developed to simulate the natural growth of tumours and their response to several treatment schemes and/or schedules in the patient individualised context. A number of mutually compatible detailed models of specific tumour biomechanism aiming at enhancing our understanding of the natural phenomenon of cancer are used to optimise individualised cancer treatment [4].

This tool will be available in the portal later and it will contain different modules for the three main cancer branches (breast cancer, Wilms tumour, and acute lymphoblastic leukaemia) addressed in the p-medicine project. All of them will be integrated into the portal.

### 5.6 Data-mining tools

Data-mining tools allow the p-medicine users to execute scientific workflows, to set up input parameters and view results after completion. Workflows are the standard way to organise specific steps of the data-mining process (for example, preprocessing, feature generation, training, parameter optimisation, and validation) into a series of logical and standardised steps. As required by the users, p-medicine supports the execution of workflows defined by workflow environments, which are de-facto standards in scientific and biomedical computing. In particular, the execution of Taverna (open source and domain-independent workflow management system: http://www.taverna.org.uk/) workflows is supported.

**Figure 8.**
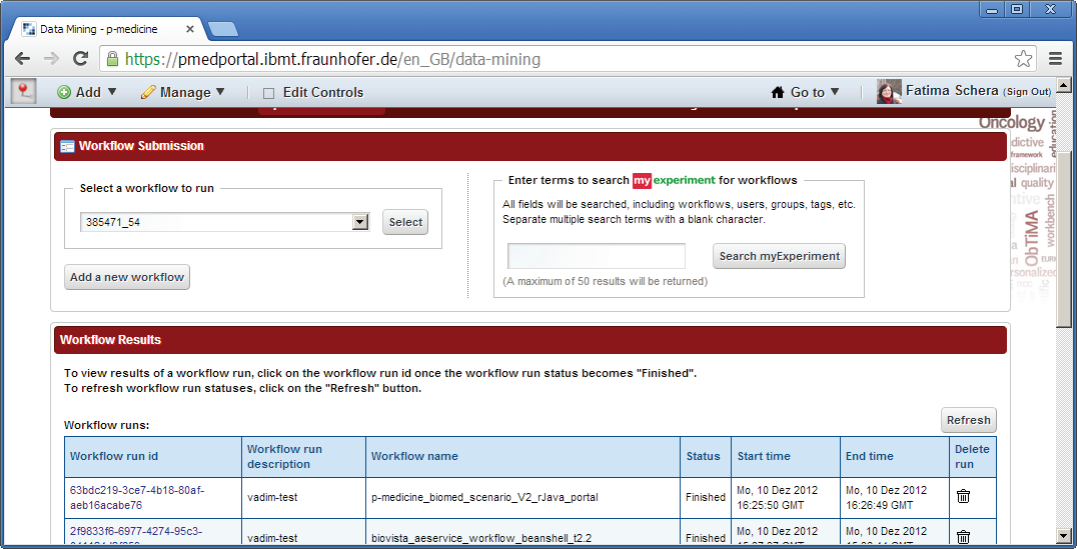
Data-mining portlets.

There are two open source data-mining portlets provided by Taverna, which have been modified for p-medicine purposes and already integrated into the portal (Figure 8):

- Workflow Submission portlet—a portlet for submission of a workflows. The portlet accesses a repository with known and available Taverna workflows.
- Workflow Results portlet—a portlet for showing results of performed workflows.

### 5.7 Clinical decision support tools

Clinical decision support (CDS) tools have been developed to enable the clinical specialists to efficiently access data and infer knowledge necessary to reach the most accurate decision for the best patient outcome. CDS tools integrated with the electronic health record systems provide a tool set to ensure that the right information is available where, when, and how clinicians need it and that clinicians follow the proper clinical processes. An integration approach into the p-medicine portal is not yet defined, but will be provided (including their source code) for downloading in the portal later.

### 5.8 Patient empowerment tools

The Interactive Empowerment Service (IEmS) has been developed in p-medicine to enable patients themselves to be participants of the p-medicine platform. The IEmS allows patients to manage their own health care and decide at any time what kind of research is allowed to be done with their data and their own biomaterial [5].

Patients and doctors can access the IEmS through the p-medicine portal. Then, questionnaires and intelligent profiling mechanisms are used in order to construct patient profiles. Because of collaboration among physicians, psychologists, and ITs, the patient’s profile can be combined with the patient’s health record system. These tools will be available in the portal in a later period of the p-medicine project.

### 5.9 Push services

In order to make use of the clinical data stored in the electronic clinical records in heterogeneous HIS and other biomedical databases, p-medicine provides **push services** for the seamless integration of data retrieved from HIS into the p-medicine’s data warehouse. These services integrate the data semantically by means of the HDOT ontology and the tools of the semantic layer so that the data in the data warehouse can be stored in a form compliant with this ontology.

Two kinds of scenarios are supported:

- Pushing data from external data sources into the data warehouse using a user-friendly portlet in the p-medicine portal. This tool is already developed, but not yet integrated in the portal.
- Pushing data from ObTiMA into the data warehouse (see Section 5.5). This functionality is already integrated in ObTiMA.

### 5.10 Ontology aggregator

The ontology aggregator tool is a tool for aggregating semantic resources in an (semi-) automated fashion under HDOT. This tool allows users to find and select relevant parts of semantic resources and semi-automatically integrate those parts under HDOT, i.e. users can reuse preexisting semantic resources and extract classes or terms from them to design specific HDOT modules for their specific needs. These tools will be available in the portal in a later period of the p-medicine project.

### 5.11 Ontology annotator

**Figure 9.**
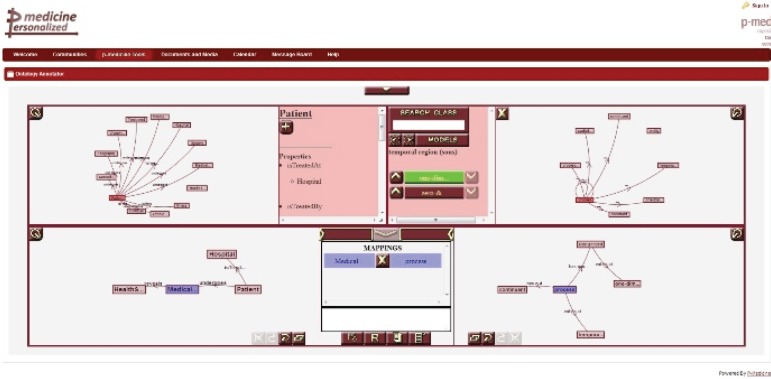
The ontology annotation tool.

The **ontology annotator** is an external web-based graphical tool already integrated as iframe portlet (Figure 9) in the p-medicine portal, which allows end users (specifically data managers) creating the necessary annotations for the translation of data in the databases integrated in the data warehouse into HDOT format. The tool provides a graphical representation of the schema of the database integrated in the p-medicine platform and HDOT.

### 5.12 Custodix Anonymisation Tool Services

The CATS (https://www.custodix.com/index.php/products/data-privacy/cat) is a service responsible for the de-identification of clinical data files (including imaging data). CATS de-identifies data files based on their mime type, schema and a set of preconfigured transformation rules (privacy profiles).

CATS scans for patient identifying information. This identifying information can be cleared or replaced by a pseudonym. The reference between the patient and the pseudonym can be stored locally or on patient information management system (PIMS; https://www.custodix.com/index.php/products/data-privacy/pims). PIMS is a tool that stores patient/pseudonym references and matches patient records coming from different sources. CATS can also encrypt (parts of) the result file to keep sensitive information confidential.

This application can be later started from the portal but currently it runs locally at the data source (e.g., the clinician’s laptop).

### 5.13 Data warehouse access tools

Data Warehouse is a central research repository of p-medicine with respective services for collecting, sharing, and further elaborating annotated anonymised clinical data and other research relevant data from diverse heterogeneous sources, such as in particular clinical trials and electronic patient records from HIS.

The data warehouse will provide a HTML-based user interface integrated into the p-medicine portal for browsing, based on terms of the HDOT ontology, and advanced querying of the structured data using SPARQL (a recursive acronym SPARQL protocol and RDF query language; http://www.w3.org/2009/sparql/wiki/Main_Page) on one or more instances of the p-medicine data warehouse. These data will include results from clinical trials, records from private institutions databases, and even information available from public biomedical repositories. In order to provide a homogenised access over all these data, all handled records will be transformed into an HDOT compliant form at the moment of loading them in the data warehouse using so-called data translation services. These services are invoked whenever any data are pushed into the data warehouse, and perform a dynamic translation of all registries being pushed. These services consider coherence of data in order to correctly realise merging of similar data and transformation of literals. The data translation service operations are performed in a transparent manner, hiding the details from the users that perform the data push.

The data warehouse and the data translation services are accessible via ObTiMA and data-mining tools in the portal, but the portlet for browsing the data warehouse will be available in a later period of the p-medicine project.

### 5.14 OpenStack object storage

The data warehouse is a storage backend for files using cloud storage in the p-medicine environment. The other scenarios are related to data-mining workflows that use cloud storage for intermediate computation results (Section 7.1), and oncosimulator application executed in a dedicated cluster environment (Section 5.5). The p-medicine cloud storage system is built based on open source OpenStack Object Store Swift (http://swift.openstack.org/) cloud storage technology. It provides access to reliable storage space considering requirements from different end-user scenarios: long-term data preservation on the one hand, as well as fast access to application data in the workflow execution.

For storing medical imagery, the open source Digital Imaging and Communications in Medicine (DICOM) server for the health-care enterprise dcm4chee (http://www.dcm4che.org/) is integrated directly with the OpenStack Object Store service to extend storage space for large and numerous medical images, which could be added to the system. DICOM is the de-facto standard for handling, storing, printing, and transmitting information in medical imaging.

For quick and precise identification and delineation of tumours in medical images, the Dr Eye (http://biomodeling.ics.forth.gr/) DICOM cancer image analysis and visualisation platform will be used.

This tool is already integrated in the portal.

## 6 Security framework of the p-medicine portal

The p-medicine platform provides a lightweight dynamic security architecture, which is already integrated in the portal. It consists of modular reusable components, which deal with security problems, such as authentication, authorisation, auditing, and de-identification. Those components are based on commonly used industry standards, such as Security Assertion Markup Language (SAML) (an OASIS standard that defines an XML-based protocol, making it possible to exchange authorisation and authentication data within or between security domains: http://saml.xml.org/about-saml), Web Service (WS) [e.g., WS-Security (https://www.oasis-open.org/committees/tc_home.php?wg_abbrev=wss), WS-Trust (http://docs.oasis-open.org/ws-sx/ws-trust/200512/ws-trust-1.3-os.html), and XACML (XSAML is an XML-based declarative access control policy language defining both a policy, decision request and decision response language. It is based on the attribute-based access control model, which incorporates role-based access control: http://docs.oasis-open.org/xacml/3.0/xacml-3.0-core-spec-os-en.pdf).

**Figure 10.**
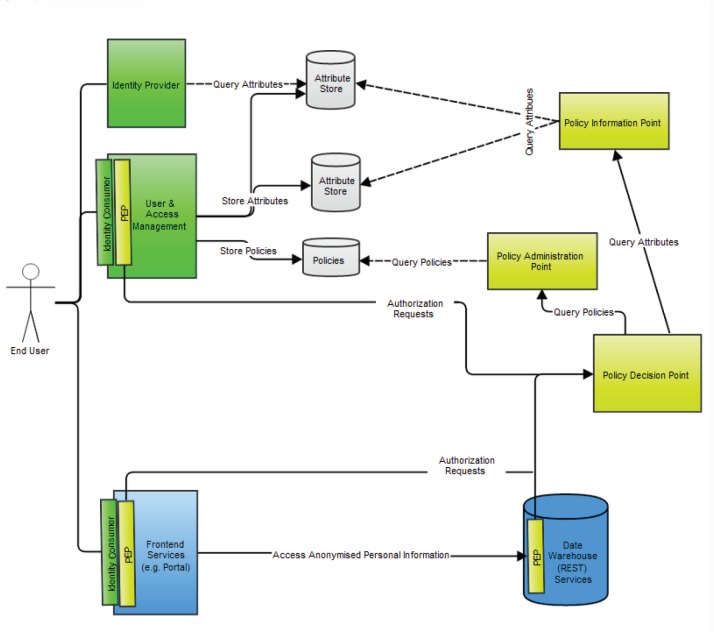
An overview of the p-medicine security architecture.

Major authentication components in the p-medicine security architecture are (Figure 10):

- The identity provider (IdP) that is a service provider within the security infrastructure responsible for authentication. It provides identity assertions to other service providers. The IdP is based on the SAML 2.0 with the open source Shibboleth (http://shibboleth.net) as implementation.
- An identity consumer, which is a software component that is part of a service provider. It consumes the SAML assertions provided by the IdP. It verifies the received assertion and passes it to the service provider’s application layer.
- A user enrolment and management service where users can be enrolled, revoked, edited, and so on.
- A secure token service responsible for issuing SAML identity and delegation tokens for representational state transfer (REST: http://www.ibm.com/developerworks/webservices/library/ws-restful/) and Simple Object Access Protocol (SOAP: http://www.w3.org/TR/soap/) clients. REST and SOAP are protocols intended for exchanging structured information in a decentralised, distributed environment.

Authorisation within the framework is based on the XACML. Major authorisation components in the architecture are:

- An XACML Policy Enforcement Point, which is a software component that requests and enforces authorisation decisions.
- An XACML Policy Decision Point (PDP), which is an entity that makes authorisation decisions. A PDP accepts authorisation requests and makes a decision based on policies fetched from a Policy Administration Point (PAP).
- A Policy Information Point (PIP), which is an endpoint, that provides missing information to a PDP, i.e. attribute information. For example, if a policy requires information on a specific attribute that has not been provided within the authorisation request, a PDP requests a PIP for information on that attribute.
- A PAP is an endpoint that manages XACML policies. It provides a PDP with all policies required to produce an authorisation decision.
- An Authorisation Rule (Policy) Management Service where authorisation rules can be configured generating XACML authorisation policies. It is a part of the user and access management.

Liferay integrates with the p-medicine authentication components by acting as an identity consumer of the p-medicine IdP. The p-medicine user enrolment and management services are also integrated into Liferay to provide a seamless user experience.

The p-medicine portal users do not need to sign in to every application separately. With the SAML 2.0 single sign-on functionality, provided by the Shibboleth IdP, the login procedures of different applications are centralised in one system, accessible with only one password. After entering the right password, only the applications that the user is authorised to will become available.

### 6.1 User management in the portal

The user enrolment and management service is an important component of the p-medicine security framework. It is responsible for user enrolment and user identity and credential management. The service encompasses:

- **An administration** site where user administrators register new users, manage existing users, manage and create organisations, manage organisation membership, and enable, disable, and remove users.
- **A user profile** page where users manage their own identity attributes, such as first name, last name, and email address.
- **A public registration page** where users register themselves. A user who registers himself through the public registration pages first needs to be accepted by a user administrator before he can create credentials.
- **An activation page** where users choose a username and password. After a user is registered and, in case of public registration, accepted by an administrator, the user receives an activation mail. Through a link in this mail, the user is guided to the activation page where he can chose his username and password.
- **Credential management pages** where users request username or password recovery.

This user management functionality is already integrated into the portal through direct links (e.g., for username/password recovery) and iframe portlets. As user management and organisation, management is then handled by those portlets, which render specific functionality of the p-medicine user management service, the corresponding already existing original functionality in Liferay is disabled. All changes to the central p-medicine identity are automatically propagated to Liferay during authentication.

## 7 Usage scenarios

Below, we introduce some typical usage scenarios illustrating the services working together.

### 7.1 Data mining

A typical user scenario that can be conducted in the p-medicine portal is executing a scientific workflow, viewing its results, and storing the results in the p-medicine data warehouse for later reuse.

For this scenario, the portlets *workflow submission* and *workflow results* (Figure 8) are used. The data-mining service reuses algorithms encoded in the statistical language R (http://www.r-project.org/), and a workflow engine allows the users to profit from the plenitude of existing workflows shared on public workflow repositories.

For executing a workflow, the user needs to select the portlet ‘*workflow submission*’. This portlet accesses a repository with known and available Taverna workflows. To enact a workflow, the user needs to select a workflow and create a workflow run description. Users can either select one of the workflows existing in the repository using the drop-down list or upload new files with workflow definitions into that repository. In order to select a workflow for execution, the user can also use the *myExperiment* (http://www.myexperiment.org/workflows/3654.html) tool integrated in the portlet, which provides the user an access to a public database of common workflows.

After selecting a workflow, the user is obliged to set up input parameters if necessary and to start running the selected workflow. The status of the workflow execution (*finished* or *running*) as well as the start and end time are displayed in the table row for the selected and executed workflow.

**Figure 11.**
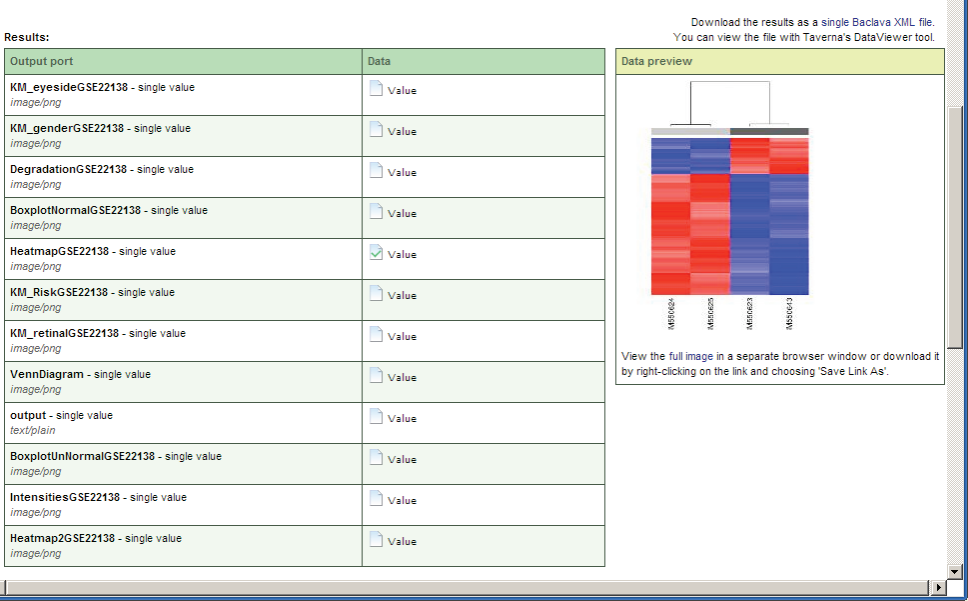
A preview of a value of the executed workflow result.

The results of the workflow enactment will be saved in a customisable system path represented as a selectable link with a unique workflow ID, which can be accessed by the *workflow results* portlet or any other software or human. A list with result values will be shown if the workflow in the *workflow results* portlet is selected. For displaying the result values, they can be selected and opened as previews or as full views using a corresponding link (Figure 11). Intermediate computation results of data mining workflows execution can use cloud storage of the data warehouse.

### 7.2 Reusing of data from HISs in ObTiMA and providing clinical trial data for research

A typical user scenario in the context of clinical trials is to create a trial, conduct a trial, and share the trial data in the p-medicine data warehouse for further analysis. That can be conducted in the p-medicine portal as follows:

1. A trial chairman creates a clinical trial in ObTiMA.
2. The trial chairman designs HDOT-ontology-based CRFs in the trial for collecting clinical data using an annotation module integrated in ObTiMA.
3. The trial chairman involves a local physician in the trial.
4. The local physician enrols patients into the trial according to inclusion/exclusion criteria defined during the trial design and fills in the patient’s CRFs with clinical data.
5. The local physician synchronises (actualises) the patient’s clinical data with data collected from HIS using the p-medicine sync services, if new data records for the patient are available. That means that data need to be entered only once in the HIS and then the data are automatically transferred to ObTiMA. The sync services, therefore, facilitate clinical research by enabling the entry of data once for research and health care and maximise the use of information from health care for the research benefit of the population. The trial chairman pushes the ontology annotated clinical data of the trial to the data warehouse using the push services integrated in ObTiMA. In the background, the data will be pseudonymised using CAT. For storing the data in the data warehouse, they will be automatically translated from the ObTiMA database format to a format compliant to HDOT using the data translation services (Section 5.13). These services require two input files, a ‘data file’ and a ‘mapping file’ for the selected trial. Since the data are semantically annotated in terms of the HDOT ontology, they can be seamless reused by the various analysis tools and services of the p-medicine platform, as e.g., data mining, simulation, and decision support tools.

### 7.3 Biobanking

p-medicine’s biobank access framework provides access to different kind of human biomaterials and related data for research purposes. Based on a minimum data set, the framework harmonises the data and enables flexibility of data import through its metadata repository. In particular, the p-medicine biobank framework will support the following user scenarios.

#### Offering biomaterial for research

A biomaterial owner can offer his biomaterial and related data to open or closed research communities, according to legal aspects. To do this, the biomaterial owner should upload his biomaterial data into p-BioSPRE from the so-called p-biobank wrapper, a local server installed at the site of a biomaterial owner by performing the following steps:

1. A biomaterial owner imports data from his biobank management system (e.g., ObTiMA), in which data are stored that he wants to share into his p-biobank wrapper. If biomaterial data for the same patient are imported from different biobank management systems into a p-biobank wrapper, the data are still linked, since for a patient always the same pseudonym is used.
2. In the p-biobank wrapper, the biomaterial owner is able to specify which data of the biobank management system will be integrated in the p-BioSPRE metabiobank and, which research communities will get access to it. This assures that the biomaterial owner keeps any case a control of his material and data.
3. The biomaterial owner uploads the data from the p-biobank wrapper to the p-BioSPRE metabiobank. The pseudonymised data in the p-biobank wrapper will be anonymised. The information that the data belongs to the same patient is preserved when the data are anonymised locally over the p-biobank wrapper and uploaded into p-BioSPRE.

The role model of the p-biobank wrapper includes the roles of a biobank/user for import, export, and search data as well as an administrator for deleting data from database, adding p-biobank wrapper users, and adding biobanks.

#### Searching and requesting biomaterial for research in p-BioSPRE

The metabiobank p-BioSPRE provides researchers the possibility to search for and request biomaterial that is offered within his communities and fits their research purposes. p-BioSPRE is a web application and database architecture, which can be accessed via the p-medicine portal. The application’s management of user roles and rights is compliant with the p-medicine security framework.

For searching and requesting biomaterial, a researcher has to perform the following steps:

1. The researcher accesses an interactive search tool (Figure 7). He selects a localisation of an organ or an organ system from which biomaterial is derived, an ICD-classified disease, a specimen type (e.g., whole blood, serum, tissue, DNA, RNA, etc.), an annotation (patient age, sex, clinical information, and genetic subtypes), information on patient’s informed consent given for the underlying biobank/trial, or he enters free text for searching.
2. As a first step search result, the researcher will retrieve the number of cases/specimens matching their request, enabling them to decide if further query will make sense.
3. If the researcher wants to go for further information on the retrieved material or request material for a research project, he may enter a project request online. For this purpose, an xls-file will be generated from the search criteria he has collated when browsing p-BioSPRE.
4. The researcher submits a request for biomaterial. The file, generated in Step 3 will be attached to the request form. The request will be transferred to the biomaterial owner for handling.
5. The biomaterial owner can decide to contact the researcher to provide the material for his research.

## Conclusions

The p-medicine portal is a web application providing comfortable access for the p-medicine users to the tools and services integrated into the p-medicine environment.

The portal has been developed following the requirements of the user groups and their roles for the p-medicine portal. The actual version contains the initial functionality of the p-medicine security framework, data-mining tools, biobank access framework, workbench, and ontology annotation tool as well as ObTiMA. Other tools and services will be integrated during the course of the project as they become available.

The users of the p-medicine portal can use available p-medicine applications in one single secure environment: they can design and run clinical trials by capturing all patient data on ontology-based CRFs, execute scientific workflows for data-mining process, simulate the response of clinical tumours to several treatment schemes as well as share human biomaterial and related data for research purposes.

The demonstration version of the p-medicine portal can be accessed using the URL https://pmedportal.ibmt.fraunhofer.de.

## Conflicts of interest

The authors have declared that no competing interests exist.

## Authors’ contributions

All the authors wrote the manuscript and agreed on the final version.
